# Clinicopathological features of primary tauopathies: a brain bank series

**DOI:** 10.3389/fneur.2026.1768959

**Published:** 2026-03-30

**Authors:** Brigid Ryan, Laura Marriott, Helen Murray, Richard L. M. Faull, Clinton P. Turner, Maurice A. Curtis

**Affiliations:** 1 Department of Anatomy and Medical Imaging, Faculty of Medical and Health Science, University of Auckland, Auckland, New Zealand; 2 Centre for Brain Research, Faculty of Medical and Health Science, University of Auckland, Auckland, New Zealand; 3 Anatomical Pathology, Department of Pathology and Laboratory Medicine, Auckland City Hospital, Auckland, New Zealand

**Keywords:** clinicopathological correlation, frontotemporal dementia, post-mortem pathology, primary tauopathy, progressive supranuclear palsy

## Abstract

**Introduction:**

This study presents the first comprehensive clinicopathological analysis of primary tauopathies from the Neurological Foundation Human Brain Bank (NFHuBB). Primary tauopathies are a heterogeneous group of neurodegenerative disorders, defined by abnormal tau protein deposition as the core pathological feature. They are further defined by the absence of an additional pathological driver. Historically, they were often accepted into brain banks as Alzheimer’s, Parkinson’s, or dementia cases.

**Methods:**

Here, we retrospectively examined all brain donations to the NFHuBB from 1994 to 2022 to identify cases of primary tauopathy, excluding those with known secondary causes such as repetitive head trauma. This is a national-level, single brain bank series. Detailed clinical records, demographic data, and post-mortem pathological investigations, including immunohistochemistry, were analysed to establish clinical and pathological diagnoses according to current criteria.

**Results:**

Fifteen cases were identified, spanning argyrophilic grain disease, primary age-related tauopathy, corticobasal degeneration, genetic frontotemporal dementia, and progressive supranuclear palsy. The mean age of onset was 69 years, with average survival of 6 years. Notably, only 47% of cases showed concordance between clinical and pathological diagnosis, underscoring diagnostic challenges. Neuropathological review revealed marked diversity in tau pathology and regional vulnerability, with most cases demonstrating both neuronal and glial tau pathology.

**Discussion:**

This case series highlights the complexity and heterogeneity of primary tauopathies and the critical role of post-mortem neuropathological examination for diagnosis. By making fixed and frozen tissue, and clinical data available for national and international dissemination, this study provides a valuable resource to advance global research into the pathogenesis and classification of tauopathies. Furthermore, this work highlights the important role of brain banking in the study of rare diseases.

## Introduction

Tauopathies are a heterogeneous group of neurodegenerative diseases that are characterised by abnormal tau protein deposition in the brain. Alzheimer’s disease is the most prevalent tauopathy. Tauopathies in which tau deposition is the predominant pathological feature are defined as “primary tauopathies”; those, such as Alzheimer’s disease, that are considered to have an additional driving force are defined as “secondary tauopathies” (1). Primary tauopathies are relatively rare, and studies of human post-mortem tissues are limited. This is partly due to the lack of banked tissue relative to more prevalent neurodegenerative diseases (2). Therefore, additional primary tauopathy cases are needed for autopsy studies to better understand anatomical vulnerability, disease progression, pathogenesis, and cellular and pathological heterogeneity of tauopathies (3). Comparative studies are useful to interrogate the drivers of cell-type specificity of tau aggregation, particularly in light of recently developed tau-targeting treatment approaches, which are likely to be specific to individual tauopathies (1).

Human brain banking is one of the central ways researchers can study the neuropathology of tauopathies. Many patients with tauopathies have extensive clinical information due to the complexity of the conditions and the slow and methodical way in which they are diagnosed. Identifying and pooling primary tauopathy cases from multiple brain banks may be necessary to perform sufficiently powered studies. Furthermore, brain banks can be the link between researchers and families with specific conditions leading to wider studies of rare familial disease. For example, researchers in New Zealand established “The New Zealand Genetic Frontotemporal Dementia Study (FTDGeNZ)”. The study began with donation of the proband’s brain to the Neurological Foundation Human Brain Bank (NFHuBB) and subsequently 27 family members have been studied to identify the earliest changes in pre-symptomatic disease, illustrating the value of banking even individual cases of rare diseases.

The NFHuBB was established in 1994 to collect the brain and associated tissue from donors with neurologic diseases and neurologically normal individuals. At the NFHuBB we have received brain donations of rare disease since our inception, but most studies utilising brain tissue from our bank are focused on more prevalent diseases such as Alzheimer’s or Parkinson’s disease. However, it is imperative that brain banks also publicise and make available for researchers, the rare disease cases they hold as they may hold the secrets to understand more common diseases and are important diseases in their own right. Here we sought to identify and characterise all primary tauopathy cases in the NFHuBB as a subset of primary tauopathy cases in New Zealand, and to make them available for collaborative research studies. Control tissue from neurologically normal individuals is also available from the NFHuBB.

Primary tauopathies are clinically, biochemically, and morphologically diverse, and the underlying disease mechanisms are largely unknown. Primary tauopathies are categorized as distinct pathological phenotypes based on the anatomical location of tau deposition, affected cell-type/s, and the tau isoforms that are included in pathological aggregates (3). In all primary tauopathies, hyperphosphorylated tau is the major constituent of neuronal and/or glial inclusions. Clinically, primary tauopathies present as dementias (most commonly frontotemporal dementia) and/or movement disorders. There is a complex interaction between the pathological and clinical phenotypes of primary tauopathies; they are best understood as clinicopathological entities, consisting of the underlying pathological phenotype and the resulting clinical syndrome (4). Furthermore, primary tauopathy nomenclature overlaps with that of frontotemporal lobar degeneration (FTLD), specifically the FTLD-Tau subset. The complicated relationships between clinical and pathological phenotype pose significant challenges for accurate diagnosis and the gold standard remains post-mortem neuropathological examination. Recent advances in *in vivo* detection of tau in the blood, cerebrospinal fluid, and via neuroimaging hold promise to improve diagnosis of primary tauopathy (5), and post-mortem studies will be critical to validate the sensitivity and specificity of these techniques (1).

Previous brain bank-based series of tauopathy cases have identified and/or characterised cohorts of progressive supranuclear palsy (PSP) (6), corticobasal degeneration (CBD) (7), and argyrophilic grain disease (AGD) (8), for example. These studies have contributed significantly to the field by shedding light on the classification of primary tauopathies and elucidating their pathogenesis.

Here we have identified and characterised all primary tauopathy cases from the NFHuBB, establishing a cohort of cases that had been largely understudied and seldom requested for research. A key advantage of this brain bank series is the extensive clinical records that are available for many of the cases banked in the NFHuBB, allowing clinico-pathological correlation for individual cases. By detailing their key pathological features and making this information publicly available, we aim to facilitate the use of these tissues and associated clinical data to advance understanding of primary tauopathies.

## Methods

### Case identification

NFHuBB is the only brain bank in New Zealand. Since 1994 it has received brain donation from over 800 individuals. All cases received at the NFHuBB between January 1994 and December 2022 were screened for inclusion in this case series. The NFHuBB categorises cases based on the clinical diagnosis at acceptance; for example, if a patient that donated their brain was clinically diagnosed with Alzheimer’s disease, this case would be assigned the code AZ, followed by an identifying number (PD – Parkinson’s disease, FTD – Frontotemporal dementia, HC – Huntington’s disease). Once preserved, cut, and stained by our brain bank staff, cases are sent for pathological analysis. If the pathological diagnosis differs from the clinical diagnosis, the original NFHuBB code remains the same; however, the pathological diagnosis will be recorded. Prior to 2020 any change of diagnosis was only recorded on a paper copy of the pathological report. Therefore, we manually inspected pathology reports prior to 2020. We restricted the search to cases designated with AZ, PD, HC and FTD case codes. These disease subgroups were chosen because primary tauopathy cases were most likely to have been accepted into the NFHuBB with these clinical diagnoses. In addition, we screened all cases that were accepted into the NFHuBB as neurologically normal controls (H – Healthy), because some primary tauopathies can be asymptomatic (e.g., AGD, ARTAG, PART).

Cases were included if they had a final pathological diagnosis of any primary tauopathy. In addition, if tau pathology was identified in the pathological report of any of the screened cases, they were further investigated to determine whether they met current pathological diagnostic criteria for any of the primary tauopathies. We took this step to ensure that we identified all primary tauopathy cases, even if they were received into the NFHuBB before pathological diagnostic criteria were established [for example, the neuropathological criteria for PART were not introduced until 2014; PART was previously called “tangle-predominant senile dementia” (TPSD) or “tangle-only dementia”]. We defined primary tauopathy as any neurodegenerative disease that is characterised by abnormal tau protein deposition in the brain and is not considered to have an additional driving force. Chronic traumatic encephalopathy (CTE) was excluded, as it was considered to have an additional driving force, i.e., repetitive head trauma. We excluded cases that exhibited tau pathology but had been assigned another primary pathological diagnosis; for example, if a case had a primary diagnosis of PD with concomitant ARTAG it was excluded.

### Clinical records

Participants’ medical records were obtained from primary care and/or hospital records, and demographic and clinical information were extracted using a standardised template. All clinical records were obtained with signed family consent. Retrospective information was collected by contacting the patient’s general practitioner or requesting the records through the hospital record system and were tabulated by NFHuBB staff. Staff were not blinded to clinical or pathological diagnosis during data extraction. This includes but is not limited to: sex, weight, ethnicity, age at initial clinical diagnosis, initial symptoms related to diagnosis, any revised clinical diagnoses, age at death, cause of death, symptoms at death, familial disease profile (family member(s) with AD, PD, or other neurological conditions), and duration of disease (years from diagnosis to death). Extra information relating to their neurological status often included results from neurological tests/neuropsychiatric assessments, MRI/CT/X-ray scans, genetic testing, and involvement in any drug trials. General medical information included diabetic status, cardiovascular status, any sensory conditions, previous operations or surgeries related to neurological condition, any injuries caused by neurological status, previous concussions, medicines prescribed for neurological condition, cognitive function, emotional state, independence with daily tasks, highest education level, occupation(s), recreational drug and alcohol use, and smoking status. Data were unavailable for some cases due to incomplete clinical records and/or the number of years since the patient’s death. Initial diagnosis was defined as the first diagnosis assigned by the treating clinician. Any additional diagnoses assigned by the treating clinician were recorded as “revised” diagnoses. Disease duration was defined as years from first clinical diagnosis until death. “Post-mortem clinical diagnosis” was defined as the most likely clinical diagnosis based on pathological assessment in conjunction with clinical history.

### Neuropathological assessment

Post-mortem human brain tissue was donated to the NFHuBB with consent from donors’ families and its use in this project was approved by the Health and Disability Ethics Committee 14/NTA/08. Brains were processed and stored according to previously published protocols (9). Neuropathological diagnoses were confirmed through pathological examination of disease-specific protein aggregates by a neuropathologist, according to standard diagnostic criteria. Specimens from 8 to 18 brain areas were stained with histostains and antibodies as detailed below. In cases where the neuropathological examination revealed multiple pathologies, the pathology most consistent with the clinical presentation was designated as the primary diagnosis, with others documented as concomitant. Pathological reports were written based on the current pathological criteria at the time of brain donation; however, in some cases there has been additional pathological assessment and supplementary reporting. Here, we report all available pathological findings that are relevant to the pathological diagnosis. We also report any pathological findings that indicate concomitant pathologies. For all cases, we confirmed that the final pathological diagnosis aligned with current diagnostic criteria for the relevant primary tauopathy. Inter-rater review of pathology reports was not performed.

Pathological reports also include detailed information about the extent and morphology of the tau present within individual cases. However, the extent of this reporting varied depending on when the case was accepted into the NFHuBB, as routine tau immunohistochemistry has become more extensive over time. For example, the tau isoforms 3R and 4R have only been included in pathological assessment since 2020. Here, we summarise all available information on tau location, morphology, and isoform composition for each case.

### Pathological staging

Braak staging and Thal phase assessment are performed on NFHuBB cases where Alzheimer’s disease is suspected as a possible pathological diagnosis. Thal phase assessment is frequently utilized in combination with Braak staging to characterize the spread and burden of amyloid-β and tau pathology in the aging and Alzheimer’s disease brain. Although these pathological staging approaches are specific to Alzheimer’s disease, we report them when they have been performed for primary tauopathy cases, to provide more detail about tau pathology and comorbid amyloid pathology.

Tau Braak staging was carried out by a neuropathologist to describe the anatomical distribution of neurofibrillary tangles composed of abnormally phosphorylated tau protein within the brain. Cases with tangles confined to the transentorhinal and entorhinal cortex were classified as Stages I–II; those with involvement of the limbic regions, including the hippocampus were designated Stages III–IV; and cases with deposition in neocortical association areas were assigned Stages V–VI. In addition, Thal phases provide a five-stage system for evaluating the deposition and anatomical distribution of amyloid-β plaques. Phase 1 equates to amyloid-β deposits in the neocortex. Subsequent phases document the sequential spread of plaques into the hippocampus (Phase 2), striatum (Phase 3), brainstem nuclei (Phase 4), and finally the cerebellum (Phase 5).

### Immunohistochemistry

Immunohistochemistry was performed on formalin-fixed, paraffin-embedded brain tissue sections (7 μm) using standardized protocols. In preparation for pathological assessment, the 18 regions were stained for the pathological markers AT8 (p-tau), alpha synuclein, amyloid, and TDP-43, as detailed by (9). Since 2006, this pathology panel has expanded to include p62, 3R tau (Millipore; 05-803) and 4R tau (Millipore; 04-804). These markers cover key pathological markers for various neurological diseases and aid in the pathological diagnosis of cases donated to the NFHuBB. For each antibody, both positive controls and appropriate negative controls were included for every case and pre-treatment regimen. Antigen retrieval was performed using either Tris-EDTA buffer (pH 9.0) or citric acid/sodium citrate buffer (pH 6.0) in a pressure cooker, according to antibody requirements. A formic acid pretreatment step (10 min) was used for all antibodies except p62, and Tau-3R staining included an additional pre-incubation in potassium permanganate and oxalic acid prior to formic acid exposure.

### Histochemistry

Paraffin-embedded brain tissue sections were cut at a thickness of 10 μm and stained for myelin with Luxol fast blue. The Luxol fast blue staining solution was prepared by dissolving 1 g of Luxol fast blue in 1,000 mL of 95% ethanol (SDA) with 5 mL of 10% acetic acid, followed by filtration. Sections were counterstained with standard hematoxylin and eosin (H&E) following standard protocols, then dehydrated, cleared, and mounted. Hematoxylin staining was performed with Harris Hematoxylin for 8 min. Slides were subsequently dehydrated in 95% alcohol for 20 s before counterstaining in alcoholic eosin (Leica) for 3 min.

## Results

### Case identification

**Figure 1:**
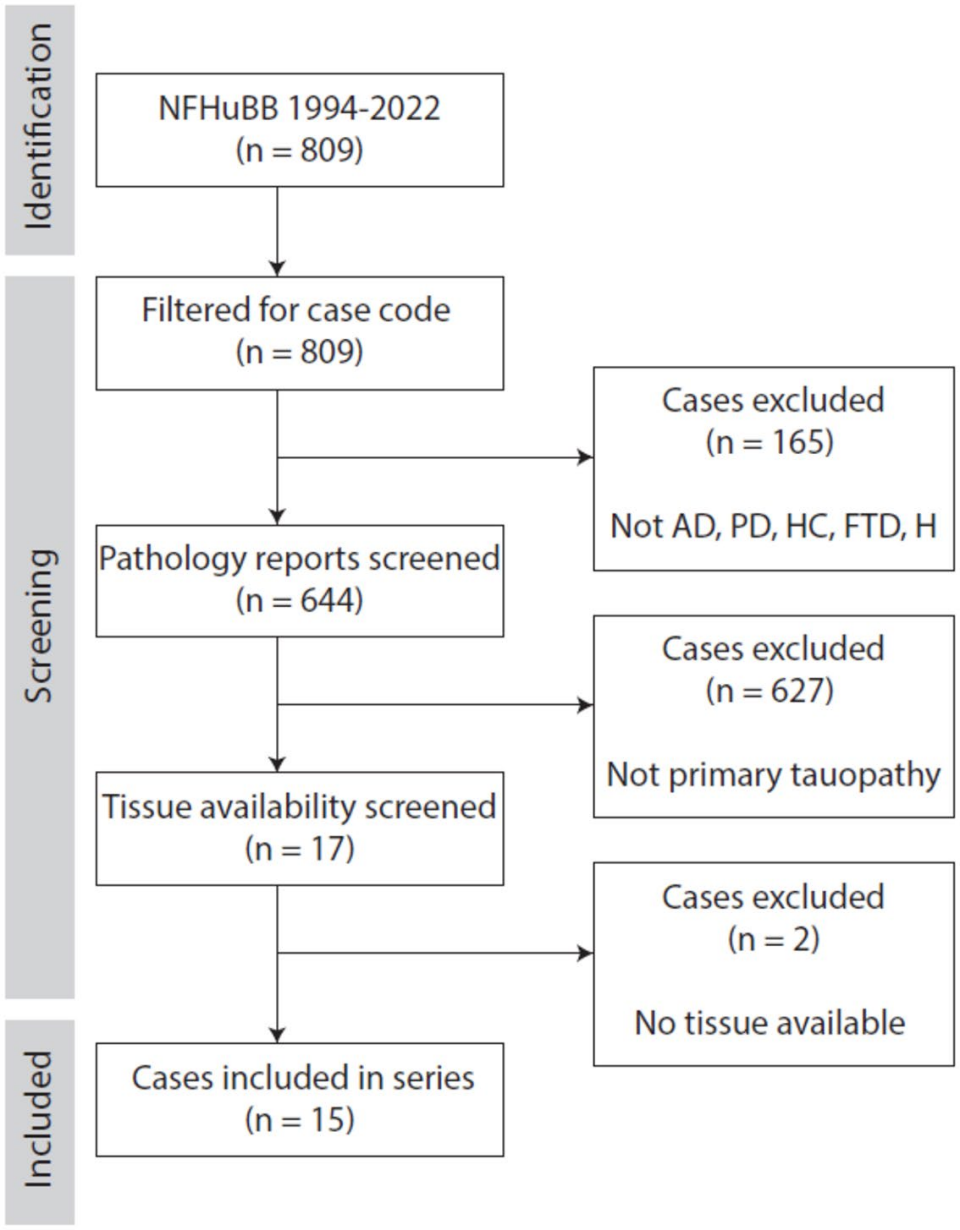
Flow diagram indicating the number of potential cases, screening process, and reasons for exclusion. AD, Alzheimer’s disease; FTD, frontotemporal dementia; H, healthy control; HC, Huntington’s chorea; NFHuBB, Neurological Foundation Human Brain Bank; PD, Parkinson’s disease.

Using pathology reports, >600 cases were screened for inclusion (Figure 1). This included cases that were donated as early as 1994 to as recently as 2022. Seventeen primary tauopathy cases were identified. We determined how much information was available for these cases (clinical and pathological), and the tissue availability. Due to the length of time since the original collection of some cases, some tissue had been discarded. Cases lacking usable tissue (*n* = 2) were excluded. We therefore included 15 cases with a pathological diagnosis of primary tauopathy.

### Demographic and clinical data

Pathological diagnoses included eight progressive supranuclear palsy (PSP) cases, two corticobasal degeneration (CBD), two genetic frontotemporal dementia (MAPT-FTD), one Pick’s disease (PiD), one argyrophilic grain disease (AGD), and one primary age-related tauopathy (PART) case. No cases of globular glial tauopathy (GGT) or tangle-only dementia were identified.

Demographic and clinical data are summarised by pathological diagnosis sub-group in Table 1. Clinically, the only two diagnoses with more than one case were PSP (*n* = 8) and bvFTD (*n* = 4). Of the 15 cases, there were 10 males and five females. The average age at the time of initial diagnosis for the whole cohort was 69 years (range: 52–86), and the average age at death was 75 years (range: 63–96). As expected, age at onset and age at death for bvFTD, PSP, and CBS were younger than for AGD and PART. Survival time varied widely, both in the cohort as a whole (mean: 6 years; range: 2–12 years) and between pathological sub-groups.

**Table 1:** Summary of primary tauopathy cases in the Neurological Foundation Human Brain Bank.

Pathological diagnosis	Number of cases	Sex (M, F)	Age at diagnosis (y)	Age at death (y)	Survival (y)	Post-mortem delay (h)	Time in storage (y)	Post-mortem clinical diagnosis, *n*
FTLD-Tau								
PSP	8	6, 2	70 (64–76)	76 (72–83)	5 (2–9)	11 (4–19)	10 (6–21)	PSP (8)
CBD	2	2, 0	71 (69–72)	73 (69–76)	4	12 (7–23)	6 (4–7)	bvFTD (1) CBS (1)
MAPT-FTD	2	1, 1	56 (52–60)	65 (64–65)	9 (5–12)	27 (6–48)	12 (5–19)	bvFTD (2)
PiD	1	0, 1	57	63	4	NR	3	bvFTD (1)
AGD	1	0, 1	85	92	7	22	8	AD-like (1)
PART	1	1, 0	86	96	10	21	6	AD-like (1)

Average data are presented as mean (range). Post-mortem clinical diagnosis is defined as the most likely clinical diagnosis based on pathological assessment in conjunction with clinical history. AD, Alzheimer’s disease; AGD, Argyrophilic grain disease; bvFTD, behavioural variant frontotemporal dementia; CBD, Corticobasal degeneration; CBS, corticobasal syndrome; F, female; FTD-MAPT, Frontotemporal dementia with a MAPT mutation; FTLD-Tau, Frontotemporal lobar degeneration – Tau; M, male; NR, not recorded; PART, Primary age-related tauopathy; PiD, Pick’s disease; PMD, post-mortem delay; PSP, progressive supranuclear palsy.

Demographic and clinical data are further detailed for individual cases in Table 2. The earliest case in the series was received in 2004 and the most recent case was received in 2022. The available clinical data varied between cases: there were no available clinical records for one case (PSP_2), and some clinical data were missing for other cases; however, a number of cases had extensive clinical records detailing their diagnosis and disease progression.

**Table 2:** Demographic and clinical characteristics of primary tauopathy cases in the Neurological Foundation Human Brain Bank.

Pathological diagnosis	Case ID	Sex	Initial symptoms	Initial diagnosis	Age at initial diagnosis	Revised diagnosis	Post-mortem clinical diagnosis	Age at death	Depression	Alcohol	RHI	Imaging	Year received
PSP	PSP_1	M	Motor	PSP	72	None	PSP	74	Yes	—	No	None	2004
	PSP_2	M	—	—	—	—	PSP	83	—	—	—	None	2009
	PSP_3	F	Motor	CBS	76	None	PSP	82	Yes	No	No	None	2017
	PSP_4	M	Motor	PD	64	None	PSP	73	Yes	Yes	No	None	2018
	PSP_5	M	Motor, cog	bvFTD	70	PSP	PSP	72	Yes	—	No	MRI	2018
	PSP_6	M	Motor	PD	71	None	PSP	75	Yes	Yes	No	MRI	2019
	PSP_7	M	Motor	PD	69	bvFTD	PSP	76	Yes	No	Yes	MRI, PET, CT	2019
	PSP_8	F	Motor	PD	65	PSP	PSP	72	Yes	No	No	None	2019
CBD	CBD_1	M	—	bvFTD	72	None	bvFTD	76	Yes	—	—	None	2018
	CBD_2	M	Cog, dep	AD	—	None	CBS	69	Yes	—	Yes	MRI	2021
MAPT-FTD	MAPT-FTD_1	F	Cog, beh	bvFTD	60	None	bvFTD	65	No	No	No	CT	2006
	MAPT-FTD_2	M	Cog, beh	bvFTD	52	None	bvFTD	64	No	No	No	MRI	2020
PiD	PiD_1	F	Cog, beh	bvFTD	57	None	bvFTD	63	Yes	No	No	MRI	2022
AGD	AGD_1	F	Cog	AD	85	None	AD-like	92	Yes	Yes	No	CT	2017
PART	PART_1	M	Cog	AD	86	None	AD-like	96	No	Yes	No	CT	2019

Post-mortem clinical diagnosis is defined as the most likely clinical diagnosis based on pathological assessment in conjunction with clinical history. “-” indicates data were not available for this case. AD, Alzheimer’s disease; AGD, Argyrophilic grain disease; beh, behaviour; bvFTD, behavioural variant frontotemporal dementia; CBD, Corticobasal degeneration; CBS, corticobasal syndrome; Cog, cognition; F, female; FTD-MAPT, Frontotemporal dementia with a MAPT mutation; M, male; PART, Primary age-related tauopathy; PD, Parkinson’s disease; PiD, Pick’s disease; PSP, progressive supranuclear palsy; RHI, repetitive head impacts.

As expected, the PSP cases presented with motor symptoms and the cases with dementia syndromes presented with cognitive and/or behavioural symptoms. Over the course of disease progression, nine cases (60%) developed language impairment, and 10 (67%) were either clinically diagnosed with depression, or were treated for depression-related symptoms. Four cases (27%) had a confirmed history of high alcohol use and two (13%) had a confirmed history of repetitive head impacts. Imaging data were available for nine cases (60%): six cases had magnetic resonance imaging (MRI), four had computed tomography (CT), and one had positron emission tomography (PET) during diagnosis. Cognitive screening test scores (MoCA, ACE, or MMSE) were available for most cases (*n* = 11; 73%).

### Accuracy of clinical diagnosis

Of the 15 primary tauopathy cases, seven (47%) showed concordance between the initial or revised clinical diagnosis and the post-mortem clinical diagnosis (PSP_1, PSP_5, PSP_8, CBD_1, MAPT-FTD_1, MAPT-FTD_1, PiD_1; Table 2). In seven cases (47%), pathological assessment indicated that the initial or revised clinical diagnosis given during the patient’s lifetime was incorrect (PSP_3, PSP_4, PSP_6, PSP_7, CBD_2, AGD_1, PART_1). In one case (PSP_2), there were insufficient clinical data to determine the clinical diagnosis; however, this case was accepted into the NFHuBB as a “PD” case, so it is likely that this case had been misdiagnosed with PD. Of the four PSP cases, one was misdiagnosed as CBS and three were misdiagnosed as PD. CBD_2 and the AGD and PART cases were all misdiagnosed as AD.

PSP_7, in particular, illustrates the complexity of primary tauopathy diagnosis. This patient was clinically diagnosed with Parkinson’s disease aged 69 after experiencing classical Parkinson’s symptoms, including tremor, micrographic writing, and sleep disturbances. Diagnosis was complicated by a complex medical history including a traumatic brain injury at age 42. At age 72, the patient began to exhibit symptoms associated with bvFTD and his diagnosis was revised to bvFTD with associated parkinsonism. These symptoms included impulsivity and irritability. PET imaging excluded Alzheimer’s disease and vascular dementia, and revealed a complex pattern of hypometabolism suggestive of neurodegenerative processes. As the patient’s condition progressed further, symptoms consistent with PSP became more apparent, including bilateral gaze deficiency and progressive limited vertical gaze. Post-mortem pathology was consistent with his final clinical diagnosis of PSP.

### Tau neuropathology and concomitant neuropathology

Primary tauopathies are categorized as distinct pathological phenotypes based on the anatomical location of tau deposition, affected cell-type/s, and the tau isoforms that are included in pathological aggregates (3). Table 3 summarises the neuropathological features that are relevant to the pathological diagnosis, as well as any concomitant pathology. In all cases, the final pathological diagnosis aligned with current diagnostic criteria for the relevant primary tauopathy.

**Table 3:** Neuropathological features of primary tauopathy cases in the Neurological Foundation Human Brain Bank.

Pathological diagnosis	Case ID	Tau isoform	Tau lesions: cell type			Tau lesions: predominant location	Comorbid pathology		
			Neuron	Astro	Oligo		Aβ	A-syn	TDP43
PSP	PSP_1	—	NFT, threads	Yes	—	Subcortical	No	No	—
	PSP_2	—	NFT, threads	Yes	Yes	Subcortical (Braak III)	Yes	—	—
	PSP_3	—	NFT, threads, pretangles	*Tufted*	Coiled bodies	Subcortical, motor cortex	No	No	No
	PSP_4	—	NFT, threads, pretangles	*Tufted*	Coiled bodies	Subcortical, motor cortex	No	No	No
	PSP_5	4R	NFT, threads	*Tufted*	Coiled bodies	Subcortical	Thal 3	No	No
	PSP_6	—	NFT, threads	*Tufted*	Coiled bodies	Subcortical	No	No	No
	PSP_7	—	NFT, threads	*Tufted*	Coiled bodies	Subcortical, motor cortex	No	No	No
	PSP_8	—	NFT, threads	*Tufted*	Coiled bodies	Subcortical	No	Yes	No
CBD	CBD_1	4R	NFT, threads, pretangles	*Plaques*	Coiled bodies	Cortical, BG	Thal 3	No	No
	CBD_2	—	NFT, threads	*Plaques*	Coiled bodies	Cortical, BG	No	No	-
MAPT-FTD	MAPT-FTD_1	4R	NFT, threads, pretangles, ‘Pick-like’ bodies, ‘grain-like’	Plaques	Coiled bodies	Frontal, temporal, BG	Yes	-	Yes
	MAPT-FTD_2	4R	NFT, threads, pretangles, ‘grain-like’	No tufted or plaques	Coiled bodies	Frontal, temporal, BG	No	Yes	No
PiD	PiD_1	3R	NFT, threads, pretangles, *Pick bodies*	Yes	—	Cortical, BG	Thal 2	No	No
AGD	AGD_1	—	NFT, ghost tangles, *grains*	—	Coiled bodies	MTL	Thal 1	No	No
PART	PART_1	—	*NFT, threads, ghost tangles*, ‘grain-like’	None	None	MTL (Braak II)	No	No	Yes

“Tau isoform” indicates the predominant tau isoform that was present in pathological aggregates. “-” indicates this feature was not reported on for this case. Where applicable, the hallmark tau lesion for the primary tauopathy is italicized. AD, Alzheimer’s disease; AGD, Argyrophilic grain disease; Astro, astrocyte; BG, basal ganglia; CBD, Corticobasal degeneration; FTD-MAPT, Frontotemporal dementia with a MAPT mutation; MTL, medial temporal lobe; NFT, neurofibrillary tangle; Oligo, oligodendrocyte; PART, Primary age-related tauopathy; PiD, Pick’s disease; PSP, progressive supranuclear palsy; SN, substantia nigra.

The six PSP cases that were received most recently (after 2009) were reported as having tufted astrocytes, the hallmark lesion of PSP (10). The two cases that were received earlier (PSP_1 and PSP_2) were not specifically reported as having tufted astrocytes; however, prominent astrocytic lesions were noted, and the predominantly subcortical tau deposition was consistent with PSP. PSP_2 was also reported as having “background AD-like changes” due to the presence of amyloid plaques in the hippocampus and middle temporal gyrus. Similarly, PSP_5 exhibited concomitant amyloid pathology (Thal 3). PSP_8 exhibited concomitant alpha synuclein pathology: the initial stages of Lewy body disease were noted in the form of scattered Lewy neurites and Lewy bodies within the medulla.

The two CBD cases were reported as having the hallmark lesion for CBD, astrocytic plaques (11). CBD_1 was also confirmed as being a 4R-tauopathy, consistent with the CBD criteria; however, concomitant amyloid plaques were present (Thal 3). Both CBD cases were reported as having tau deposition predominantly in the cortex and basal ganglia, in keeping with a CBD diagnosis. Interestingly, despite similar neuropathology, CBD_1 presented clinically with bvFTD and CBD_2 presented clinically with CBS.

FTD caused by a tau mutation (MAPT-FTD) can be underpinned by a number of primary tauopathy pathological sub-types (PiD, CBD, PSP, AGD or GGT) (12). The two MAPT-FTD cases reported here (both from the same family with the MAPT IVS 10 + 16 mutation) did not clearly fit any of these sub-types. The tau pathology observed in MAPT-FTD_1 most closely resembled the CBD sub-type of FTLD-Tau: structures resembling astrocytic plaques were seen in the motor, temporal, and parietal cortices; tufted astrocytes were not observed; and the tau lesions were predominantly located in the cortex and basal ganglia. However, there were also scattered structures resembling Pick bodies in the motor cortex and many grain-like inclusions in the anterior hippocampus.

Similarly, the tau pathology observed in MAPT-FTD_2 most closely resembled CBD, due to the predominantly frontal and temporal location of the tau lesions, involvement of the substantia nigra, and absence of tufted astrocytes. However, no convincing astrocytic plaques were observed, and there were some grain-like inclusions in the cortex. Previously reported MAPT-FTD cases with the same mutation have been reported to resemble CBD or GGT (13). Both MAPT-FTD cases also exhibited concomitant pathology: MAPT-FTD_1 was positive for amyloid and TDP43, while MAPT-FTD_2 was negative for amyloid and TDP43 but positive for alpha synuclein.

The Pick’s disease case, PiD-1, was reported to have the hallmark ‘Pick body’ lesions and was confirmed to be a 3R tauopathy, in keeping with the PiD neuropathological criteria (14). Concomitant amyloid plaques were present (Thal 2).

The AGD case, AGD_1, exhibited the hallmark tau lesion of grains in the medial temporal lobe (8). Glial tau pathology was also present; this is rare in PART, so this case is more in keeping with AGD than PART (8, 15). Silver staining was not available. AGD was assigned as the strongest diagnosis given tau staining forming grain and comma-like structures confined to the medial temporal lobe/hippocampal region. Concomitant amyloid plaques were evident (Thal 1).

The PART case, PART-1, exhibited AD-like tau lesions, but no amyloid plaques, in keeping with the PART neuropathological criteria (15). Although “grain-like” tau lesions were described, this case did not exhibit any glial tau pathology so was more in keeping with PART than AGD. Concomitant TDP43 pathology was also noted, specifically occasional cytoplasmic inclusions within the neurons of the dentate gyrus, and scattered thread-like structures within the CA1. Tau isoform staining can distinguish PART from AGD, as AGD is a 4R-tauopathy and PART exhibits both 4R- and 3R-positive pathological aggregates (15, 16). Unfortunately, tau isoform staining was not available for the PART case or the AGD case.

## Discussion

Since 1994, the primary focus of the NFHuBB was the study of AD, PD, HD, MND, neurologically normal, and most recently sports-related brain injuries. However, through the collection of these diseases, rigorous pathological testing has identified a small population of primary tauopathies. This is the first study to systematically identify and characterise the primary tauopathy cases in the NFHuBB. We have identified a small number of primary tauopathy cases (*n* = 15) and detailed their neuropathological and clinical features in order to facilitate their use in future research projects. By making fixed and fresh tissue available for national and international dissemination, the NFHuBB will contribute to future studies of primary tauopathy classification and pathogenesis.

The majority of the primary tauopathy cases in the NFHuBB (*n* = 8) were PSP cases, two of which had been accepted into the NFHuBB as suspected PD cases. These cases have not previously been included in research studies. There are currently no treatments available for PSP, but it is increasingly a focus of clinical trials for experimental tau-directed therapies (17), so there has been renewed interest in elucidating its underlying pathophysiology. The cohort of PSP cases identified in the NFHuBB, many of which have detailed accompanying neuropathological and clinical data, represents a useful starting point for the study of the post-mortem PSP brain in the New Zealand context, and may contribute to international cohorts of PSP cases. Previous studies of PSP have benefited from combining relatively small cohorts from multiple, international banked collections. For example, Kovacs et al. (6) studied 206 cases from eight brain banks across the United States and Europe to determine tau distribution patterns in PSP subtypes.

As expected, clinical comparison demonstrated heterogeneity both within the PSP and bvFTD clinical subtypes, and between primary tauopathy sub-groups; for example, age at onset and survival varied widely. This finding underscores the importance of pairing tissue research with the accompanying clinical data. Unfortunately, some cases in this case series were lacking clinical information due to the year received. Currently, all cases accepted into the NFHuBB have data extracted from clinical records, meaning that cases will have extensive clinical data available for researchers.

We identified a single PART case, and no cases of ARTAG (aging-related tau astrogliopathy) or tangle-only dementia. This paucity of PART and ARTAG cases is likely due to our exclusion of cases with a different primary diagnosis. For example, we are aware of cases in the NFHuBB with ARTAG as a concomitant diagnosis, alongside a primary diagnosis of PD or AD. Although some primary tauopathies, including PART and AGD, can presenting asymptomatically, they can also be associated with clinical impairment (12). In our series, the only identified PART case and the only identified AGD case both presented clinically as AD-like and were clinically diagnosed during life as having AD.

Pathological assessment of the primary tauopathies in this case series confirmed that all cases meet the current pathological diagnostic criteria for the respective disease sub-type. In 47% of the cases (7/15), pathological diagnosis aligned with the clinical diagnosis made during the person’s lifetime. In the remaining cases, pathological diagnosis revealed that the person had been clinically diagnosed with a different disease (AD, PD, or CBS). In all cases, these were diseases that are known to be difficult to distinguish clinically, highlighting the significant challenges for accurate diagnosis, and the critical importance of post-mortem neuropathological examination. As expected, concomitant pathologies were common. Sixty percent (9/15) of the primary tauopathy cases identified in the NFHuBB exhibited concomitant amyloid beta, alpha synuclein, or TDP43 pathology.

To advance the study of primary tauopathies in New Zealand, and to contribute to global studies, it will be important to encourage donation of primary tauopathy cases into brain banks. Five of the cases in this case series were accepted into the NFHuBB as suspected AD or PD cases; a further 5 cases were accepted into the NFHuBB with clinical diagnoses of bvFTD. Recently, a number of suspected bvFTD and PPA cases have been accepted into the NFHuBB, so we expect that our cohort of FTD-Tau cases will increase. This raises the possibility of including NFHuBB cases of primary tauopathy in multinational cohort studies, to further our understanding of the complex relationships between pathological phenotype and the resulting clinical syndrome. Previous studies of banked tissue have been critical to the classification of primary tauopathies and have elucidated their underlying disease processes (6–8).

Studies of banked human tissue are increasingly important in light of recent advances in the field of tau biomarkers and tau-targeting therapies, as it is critical that these are validated in autopsy-confirmed cases (18). Recent human trials of tau-targeting therapies have been unsuccessful, potentially reflecting the inadequacy of nonclinical models (5). Although studies of post-mortem tissue are necessarily limited, they can provide valuable information about the specificity and sensitivity of proposed biomarkers. For example, validating novel biomarkers in autopsy-confirmed cases is necessary to complement studies of peripheral biomarkers in CSF, blood, and tissue (18). Such biomarkers are vital for clinical trial recruitment and monitoring of therapeutic response (19).

Variation of time in storage is a limitation of this case series. Some cases have been stored for over 30 years and it is possible that comparison between cases will be hampered by differing tissue quality and/or changes in storage conditions over time. There is evidence that RNA quality in human brain tissue is unaffected by storage time (20–22), but this has not been tested directly on NFHuBB tissue. Immunogenicity and protein changes with storage time were also not measured in this tauopathy cohort.

A limitation of this case series is that the routine neuropathological assessments performed on cases accepted into the NFHuBB have changed over time, so available data vary between cases as a function of the year in which they were received. For example, 3R and 4R tau have recently been included in the pathological analysis, but are not available for many of the cases reported here. Further, the lack of comprehensive pathological assessment of tau in earlier cases means that we may have missed some cases of primary tauopathy. Additional pathology assessment would be required to clarify this, but was beyond the scope of this study. Similarly, the extent of available clinical records varies between cases. Finally, we were limited by a lack of APOE genotyping for the included cases. Nonetheless, the primary tauopathy cases identified in the NFHuBB represent an important cohort of previously un-studied post-mortem cases.

## Data Availability

The raw data supporting the conclusions of this article will be made available by the authors, without undue reservation.
